# A Rare Case of Bladder Neck Obstruction Caused by Extrusion of Necrotic Tissue Following Transurethral Water Vapor Energy Therapy for Benign Prostatic Hyperplasia

**DOI:** 10.7759/cureus.85056

**Published:** 2025-05-29

**Authors:** Seiya Shiramizu, Kazunobu Aramaki, Naohiro Fujimoto

**Affiliations:** 1 Urology, Kurate Hospital, Kurate, JPN

**Keywords:** bladder neck obstruction, bph, complication, necrotic tissue, wave

## Abstract

Transurethral water vapor energy (WAVE) therapy is a minimally invasive treatment for benign prostatic hyperplasia (BPH) that typically results in coagulative necrosis without significant clinical sequelae. However, rare complications such as urinary tract obstruction due to sloughed necrotic tissue have been reported.

We report the case of a 67-year-old man with BPH who developed acute urinary retention and underwent WAVE. Although his initial postoperative course was favorable, he subsequently presented with worsening voiding dysfunction. Imaging revealed a soft tissue mass with surface calcification obstructing the bladder neck. Cystoscopy identified a calcified mass adherent to the bladder neck, and endoscopic removal of the underlying necrotic tissue resulted in rapid improvement of urinary symptoms.

This case highlights a rare but significant complication of WAVE. Clinicians should consider the possibility of bladder outlet obstruction due to necrotic tissue extrusion in patients with delayed postoperative urinary symptoms.

## Introduction

Benign prostatic hyperplasia (BPH) is a common condition among aging men and often leads to lower urinary tract symptoms (LUTS) due to bladder outlet obstruction. Transurethral water vapor energy (WAVE) therapy, also known as Rezūm™ therapy, is a minimally invasive treatment that uses thermal energy to ablate prostatic tissue, offering symptomatic relief [1]. The procedure induces coagulative necrosis within the prostate, which is typically resorbed or sloughed without clinical consequence. Clinical trials and real-world data have demonstrated the efficacy of WAVE therapy and its low incidence of complications [1-3]. However, in rare instances, extensive sloughing of necrotic tissue can occur [4,5], potentially leading to mechanical obstruction of the urinary tract. Herein, we report a rare case of bladder neck obstruction caused by the extrusion of a necrotic tissue mass following WAVE for BPH. To the best of our knowledge, this represents only the second such case reported in the literature, highlighting the need for clinical awareness of this unusual but significant postoperative complication.

## Case presentation

A 67-year-old man had been receiving medical therapy with an alpha-adrenergic receptor antagonist and a 5-alpha reductase inhibitor for BPH over the preceding five years and was receiving treatment for hypertension. In late July 2024, he developed acute urinary retention, necessitating the placement of an indwelling urinary catheter. He was subsequently referred to our hospital for surgical management. Transabdominal ultrasonography revealed an enlarged prostate with a total volume of 78 mL. Urinalysis revealed five to nine red blood cells and more than 100 white blood cells per high-power field, with no bacteria detected. Blood tests, including inflammatory markers, were unremarkable. Voiding trial was attempted following catheter removal; however, the patient was completely unable to void.

In mid-August 2024, the patient underwent WAVE for BPH. Water vapor was delivered at six sites: three injections along each lateral lobe of the prostatic urethra, approximately 3.5 cm in total length, and an additional injection targeting a protruding adenoma at the seven o'clock position of the bladder neck.

**Figure 1:**
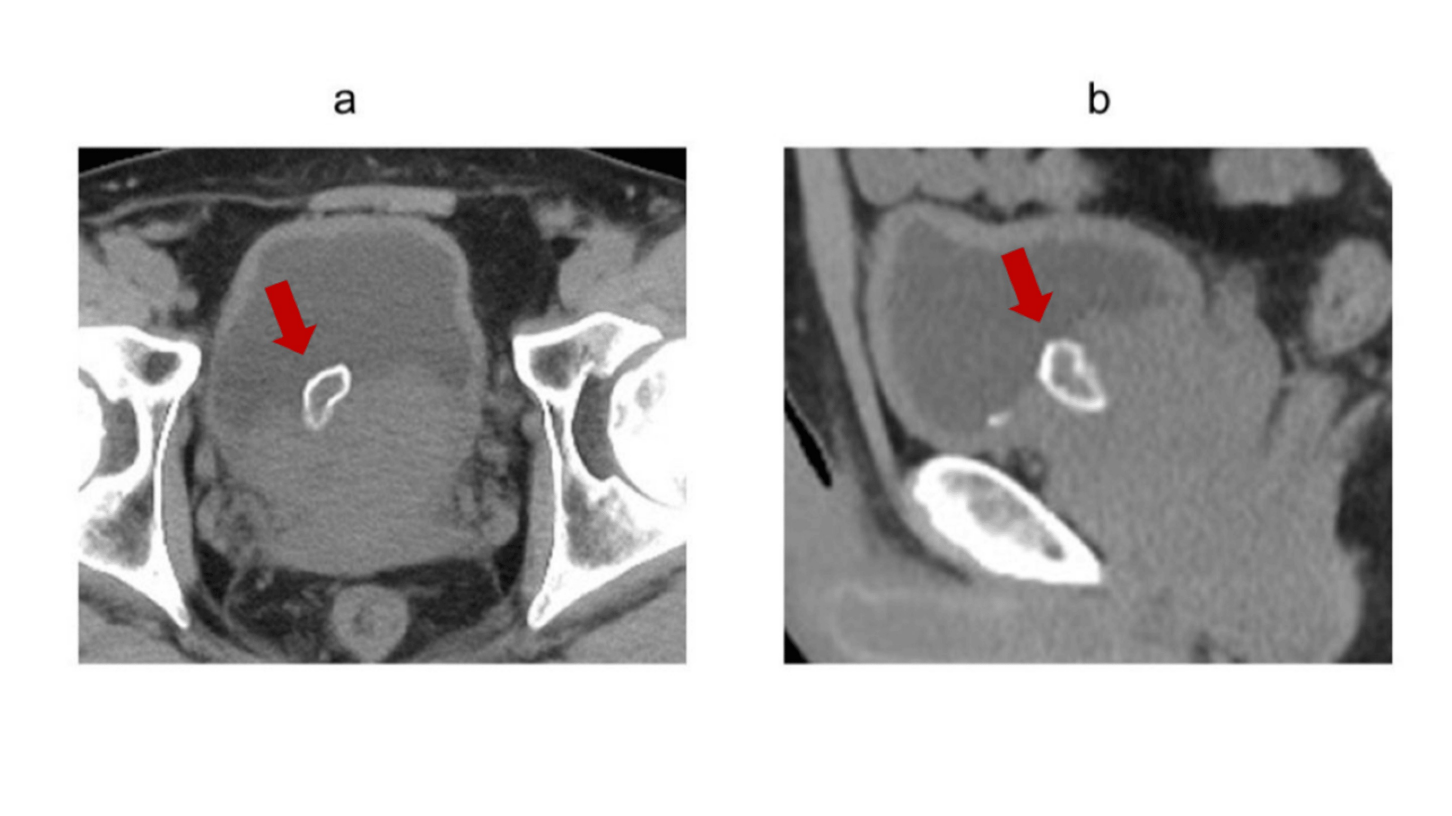
Computed tomography findings Computed tomography demonstrated a soft tissue mass with surface calcification obstructing the bladder neck. a) Axial image. b) Sagittal image.

One month after the procedure, the urinary catheter was removed, and the patient was able to void spontaneously, with marked improvement in urinary symptoms. However, approximately one month after catheter removal, he developed worsening voiding difficulty and increased urinary frequency. A post-void residual urine volume of 300 mL was measured, and computed tomography (CT) demonstrated a soft tissue mass with surface calcification obstructing the bladder neck (Figures 1a-1b).

**Figure 2:**
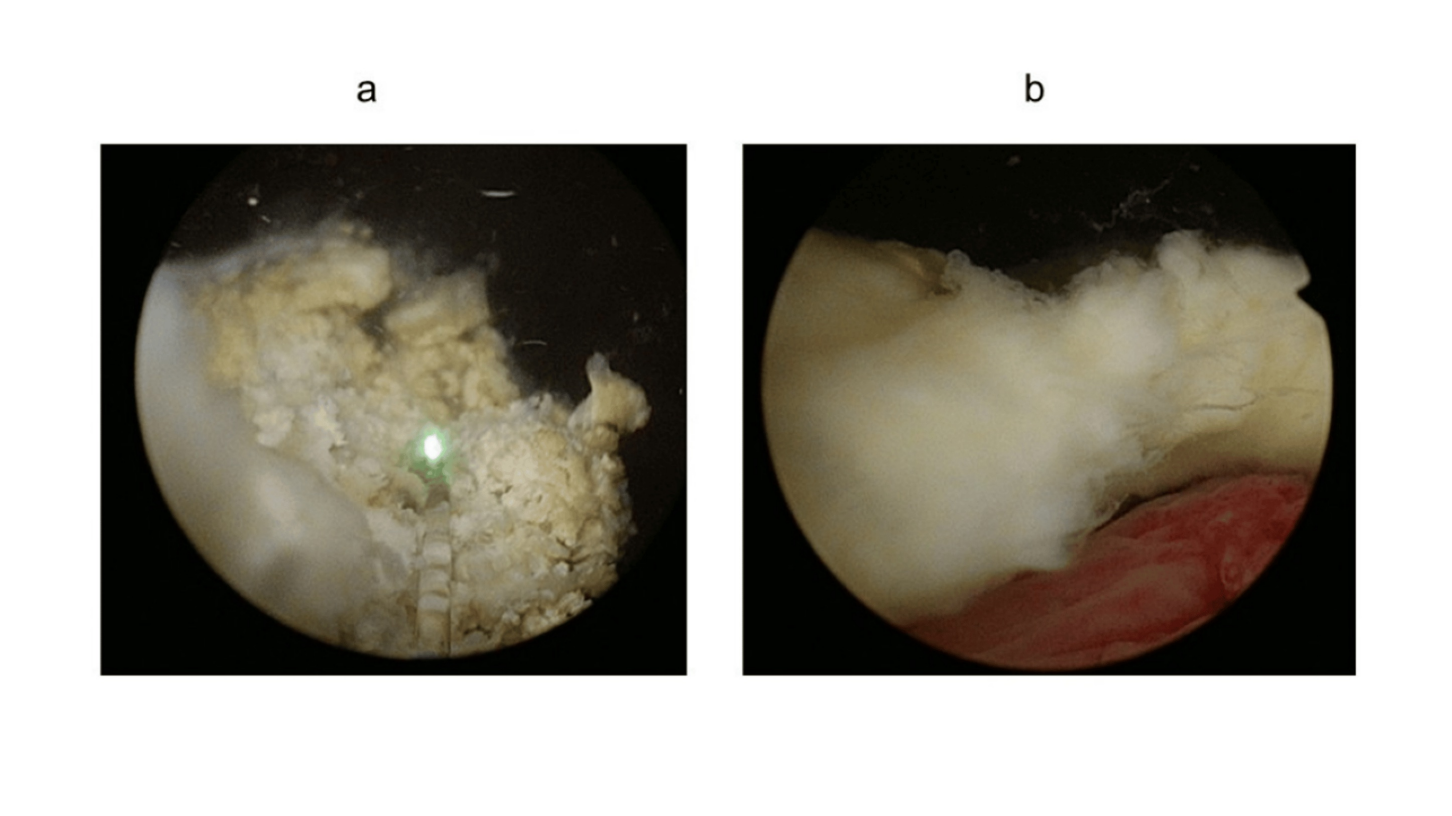
Cystoscopic findings a) A mass with surface calcification adherent to the bladder neck. b) White necrotic mass revealed after the removal of the overlying calcification.

Under general anesthesia, cystoscopy revealed a calcified mass adherent to the bladder neck at the seven o'clock position (Figure 2a), which corresponded to the site of puncture during the WAVE procedure. Laser lithotripsy was performed to fragment the surface calcification, exposing a soft, white mass underneath (Figure 2b). This mass was suspected to be necrotic tissue, and it was successfully removed using a transurethral resection loop electrode.

The urinary catheter was removed the following day, and the patient's voiding function improved significantly. At one month postoperatively, the post-void residual volume had decreased to 74 mL. At five months postoperatively, uroflowmetry demonstrated a maximum flow rate of 18.8 mL/second, his International Prostate Symptom Score (IPSS) improved to 3, and the Quality of Life index improved to 1, indicating substantial resolution of urinary symptoms.

## Discussion

In the present case, the necrotic tissue formed within the prostate following WAVE may have gradually sloughed off and migrated through the puncture tract, ultimately causing mechanical obstruction at the bladder neck. Although histologic examination was not performed, the intraoperative findings of a soft, white, non-viable mass strongly suggested necrotic tissue origin. Factors potentially contributing to this complication include the extent of thermal injury and incomplete healing of the puncture site. In our case, targeted treatment of a prominent adenoma at the bladder neck may have predisposed the patient to localized necrotic sloughing and extrusion.

Mollengarden et al. [4] reported two cases of prostate tissue sloughing following WAVE; however, detailed clinical information was not provided. Alothman et al. [5] described a case highly similar to ours, where histopathological examination of the removed mass revealed cauterized and necrotic prostatic tissue containing multiple corpora amylacea. Although histopathological examination was not performed in this case, we consider the mass at the bladder neck to be necrotic tissue based on the following reasons. First, the mechanism of WAVE induces coagulative necrosis. Second, both the endoscopic findings and clinical course in our case are consistent with those reported by Alothman et al. [5], who histologically confirmed necrotic tissue following WAVE. Finally, the mass closely resembled necrotic tissue commonly encountered in routine clinical practice, supporting its interpretation as sloughed necrotic material. Ureteral obstruction due to the sloughing of necrotic tumor cells into the pelvicalyceal system through defects in the injured pelvicalyceal wall has been reported as a complication following cryoablation therapy for renal tumors [6], and our case may share a similar mechanism.

These findings suggest that tissue sloughing with subsequent obstruction may represent a distinctive complication specific to WAVE. Given that WAVE ablates prostatic tissue through direct high-temperature water vapor injection, the mechanism of tissue necrosis fundamentally differs from that of traditional surgical interventions for BPH, potentially predisposing to the formation and extrusion of necrotic material. Voiding dysfunction in our case occurred two months postoperatively, similar to the report by Alothman et al. [5], who observed it as early as two weeks. These findings indicate that this complication may arise within two months, warranting clinical vigilance during this period.

## Conclusions

Clinicians should be aware of this potential complication, particularly in patients presenting with delayed voiding dysfunction after WAVE. Early imaging and cystoscopic evaluation are recommended if obstruction is suspected. Endoscopic removal of necrotic tissue can lead to rapid symptomatic improvement, as demonstrated in this case.
